# Supplementary figures and images for: Incomplete activation of Alyref and Gabpb1 leads to preimplantation arrest in cloned mouse embryos
Source: Life Sci Alliance. 2023 Aug 28;6(11):e202302296. doi: 10.26508/lsa.202302296 (PMC10462978; doi:10.26508/lsa.202302296)

Figure S5D  
H3K9me3

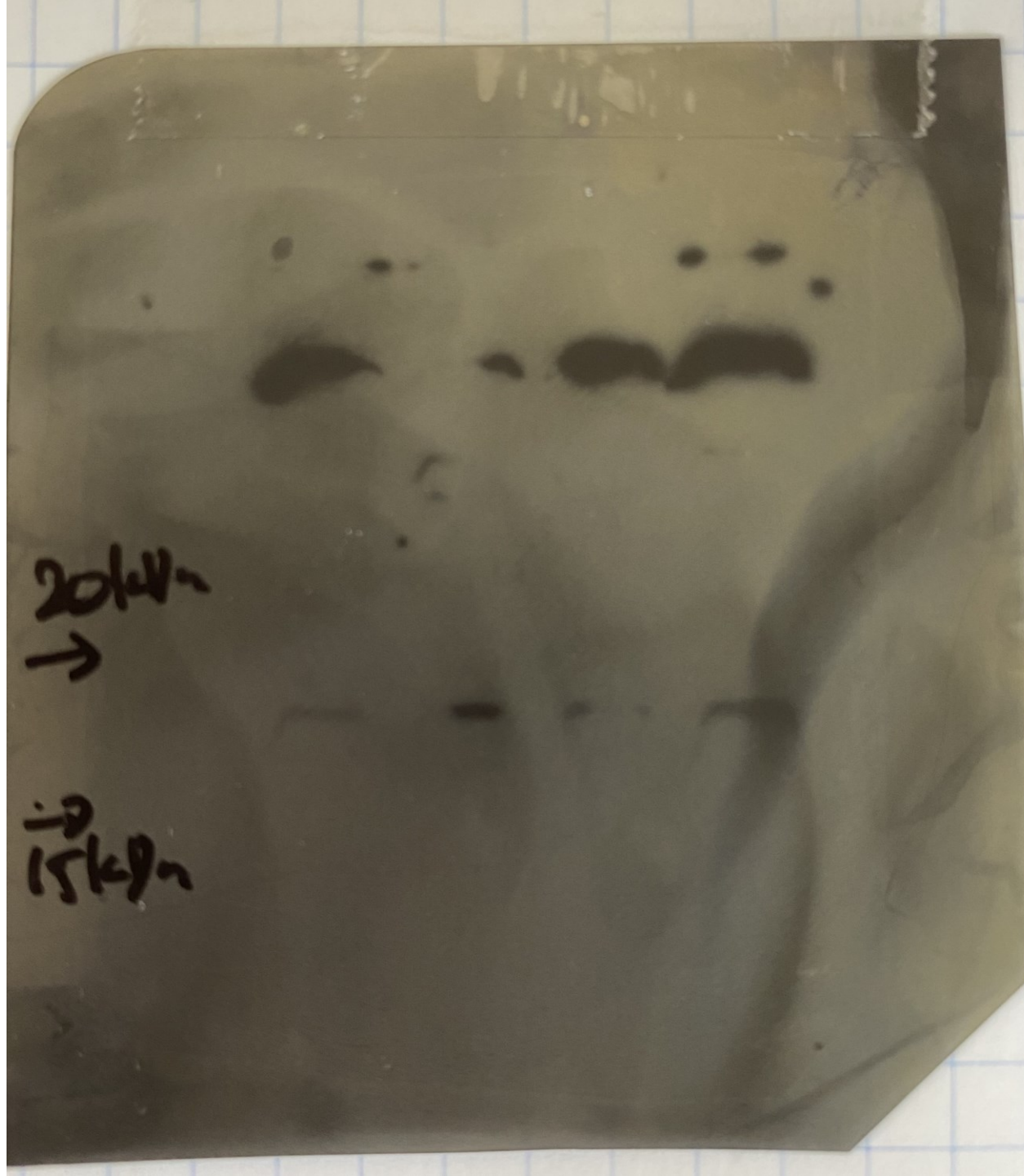

Figure S5D  
ACTB

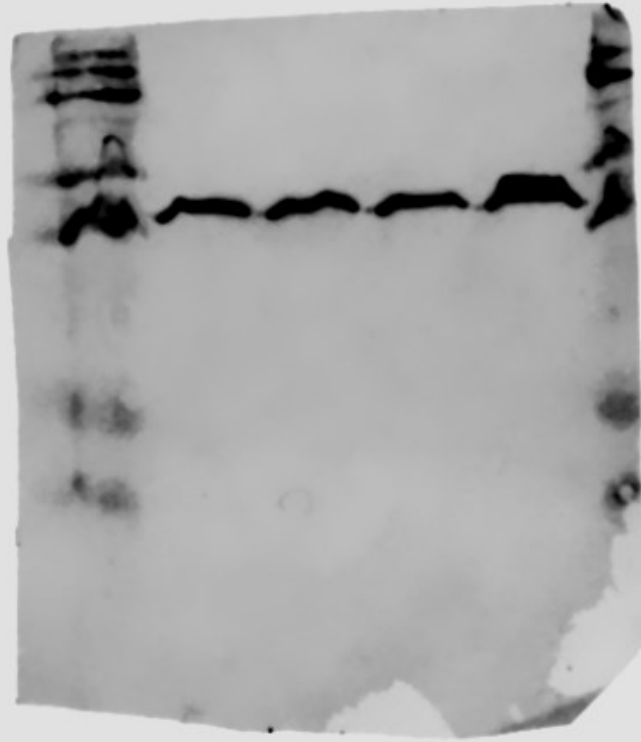

Supplement: Supplementary file 2 [file LSA-2023-02296_SdataFS5.pdf]
